# A Comparison of Cytomorphological Features of ASC-H Cells Based on Histopathological Results Obtained from a Colposcopic Target Biopsy Immediately after Pap smear Sampling

**DOI:** 10.31557/APJCP.2019.20.7.2139

**Published:** 2019

**Authors:** Hiromi Hata, Kaori Okayama, Junko Iijima, Koji Teruya, Natsuko Shiina, Timothy Caniz, Yasuyoshi Ishii, Masahiko Fujii, Mizue Oda, Mitsuaki Okodo

**Affiliations:** 1 *Department of Medical Technology,*; 3 *Department of Health and welfare, Faculty of Health Sciences, Kyorin University, 5-4-1 Shimorenjaku, Mitaka-shi, 181-8621, *; 4 *ILABO Cyto STD Laboratory, Inc., 560-6 Shimoonkata, Hachiouji-shi, 192-0154, *; 5 *Genki Plaza Medical Center for Health Care, 3-6-5 Iidabashi, Chiyoda-ku, 102-0072, Tokyo, *; 2 *School of Medical Technology, Faculty of Health Science, Gunma Paz University, 1-7-1 Tonyamachi, Takasaki-shi, 370-0006 Gunma, Japan. *

**Keywords:** ASC-H- CIN- cytomorphological features- HPV

## Abstract

**Background:**

To compare the cytomorphological features of atypical squamous cells, cannot exclude high-grade squamous intraepithelial lesion (ASC-H) observed in a liquid-based Pap smear with the histopathological features observed in a concurrent colposcopic biopsy specimen obtained immediately after obtaining the Pap smear.

**Methods:**

Cytomorphological features such as cytoplasmic differentiation, nuclear/cytoplasm (N/C) ratio, chromatin pattern, thickening of nuclear contour, and the appearance of the nucleolus of 247 ASC-H obtained from 25 liquid-based Pap smear ASC-H cases were compared with those of the cells obtained from biopsied samples. Human papillomavirus (HPV) infection was tested for 39 HPV genotypes using Uniplex E6/E7 polymerase chain reaction method.

**Results:**

Of the 25 ASC-H cases, 22 (88%) showed cervical intraepithelial neoplasia grade 1 or greater (CIN1+) and 3 (12%) were benign. HPV infection was detected in 100% CIN1+ cases and 66.7% benign cases. Significant differences such as marked hyperchromasia, thickened nuclear contour, and prominent nucleoli were observed between ASC-H cases with CIN1+ and the benign cases.

**Conclusion:**

The presence of small dysplastic cells displaying marked hyperchromasia, thickening of nuclear contour, and prominent nucleoli on Pap smear strongly suggest the presence of CIN in ASC-H cases.

## Introduction

ASC-H are divided into two main groups on the basis of cytological criteria: single and few cell groups. However, HSIL exhibits small atypical cells, and differentiating the true high-grade squamous intraepithelial lesion (HSIL) from benign reserve cell hyperplasia, endocervical glandular cells, and other benign degenerative changes has been found to be difficult in many cases, leading to a noticeable discrepancy in diagnoses among cytotechnologists and pathologists as well as among institutions (Louro et al., 2003). Thus, to improve the accuracy of the diagnosis, considerable effort has been devoted to the analysis of the cytological features of ASC-H. Cytomorphological features of ASC-H have been analyzed in previous studies using tissue biopsy specimens taken several weeks and months after the initial diagnosis of ASC-H. (Sheil et al., 1997; Montes et al., 1999; Ronnett et al., 1999; Quddus, et al., 2001; Louro et al., 2003; Selvaggi, 2003; Duncan and Jacob, 2005; Saad et al., 2006; Sherman et al., 2006; Chivukula et al., 2007; Mokhtar et al., 2008; Gupta et al., 2013a; Gupta et al., 2013b). However, it is possible that the cytomorphological features of ASC-H did not correlate with the histopathological findings in the biopsied tissue because the cells and tissue specimens were not collected simultaneously. It is plausible that analysis of cells found in the smears of ASC-H cases may have been performed on randomly selected representative cells of ASC-H judged by a cytotechnologist, or only highly atypical cells, even though the multiple characteristics of the non-selected cells could be within the criteria of ASC-H. These issues have contributed to the lack of established diagnostic criteria for ASC-H.

In this study, the Pap smears and colposcopic biopsy specimens were obtained simultaneously from ASC-H cases, allowing the cytomorphological features of the cells on Pap smears to be compared directly with the histopathological features observed in the biopsied tissue. Additionally, human papillomavirus (HPV) typing was performed on the residual cervical samples.

## Materials and Methods

*Ethical approval*

Samples were collected after obtaining written informed consent from the subjects. The study protocol was approved by the ethics committee on human research of Kyorin University (H28-27) and implemented in accordance with approved guidelines.

*Clinical samples*

Cases included in this study (n = 913) showed abnormal findings on cervical cytology and were referred for follow-up at the Genki Plaza Medical Center for Health Care in Tokyo between March 2014 and April 2018. Samples for liquid-based Pap (BD Sure Path^TM^) and punch biopsies were obtained from patients during colposcopy. 

*Cytological analysis in ASC-H cells*

The cytological parameters of the cells obtained from liquid-based Pap smears were compared with of punch biopsy findings using the cellSens^TM^ Imaging software (Olympus, Tokyo, Japan). The parameters investigated were cytoplasmic differentiation (pale or dense), chromatin pattern (marked hyperchromasia or hyperchromasia not marked or fine or coarse), nuclear/cytoplasmic (N/C) ratio (low or high), thickening of nuclear contour, and presence of prominent nucleoli.

*HPV genotyping*

The Uniplex E6/E7 polymerase chain reaction (PCR) assay, established by Okodo et al., (2018) was used for HPV typing. DNA from the residual liquid of cervical cytology specimens (100 μL) was extracted using hot sodium hydroxide and pH adjustment with a Tris solution (HotSHOT) method (Truett et al., 2000). Cell pellets were lysed with 100 μL of alkaline lysis solution (25 mM NaOH, 0.2 mM EDTA, pH 12.0) for 15 min at 95°C. Next, the lysed cells were neutralized with 100 μL of neutralization solution (40 mM Tris HCl, pH 5.0). The cells were then centrifuged at 13,200 rpm for 1 min and used directly as the DNA template in the PCR reaction mixture, which contained 1× AmpliTaq Gold^®^ 360 buffer, 2 mM MgCl_2_, 0.025 U/µL AmpliTaq Gold 360 DNA Polymerase (Applied Biosystems, Foster City, CA, USA), 1 μL DNA, and 0.5 pM primers in a total volume of 25 μL. PCR amplification was performed using a thermal cycler with 35 cycles of denaturation at 95°C (30 s), annealing at 60°C (30 s), and extension at 72°C (30 s), including an initial denaturation step of 10 min and a final extension step for 5 min. 

In the present study, HPV types were classified into two groups: high-risk genotypes (HPV-16, -18, -31, -33, -35, -39, -45, -51, -52, -56, -58, -59, and -68) (Sasagawa et al., 2016) and other genotypes including low-risk, probable high-risk, and undetermined risk types (HPV-6, -11, -26, -30, -34, -40, -42, -53, -54, -55, -61, -62, -66, -67, -69, -70, -71, -73, -74, -81, -82, -84, -85, -89, and -90). Since PCR amplification is generally prone to false-positive results, ambiguous results were re-examined.

*Statistical analysis*

Statistical analysis was performed using SPSS version 21.0 (SPSS, Chicago, IL). Between-group differences were examined using χ^2^ or Fisher’s exact probability test according to the characteristics of data distribution. Data were also complemented using adjusted residual analysis. A P value of < 0.05 was considered significant.

## Results

**Figure 1:**
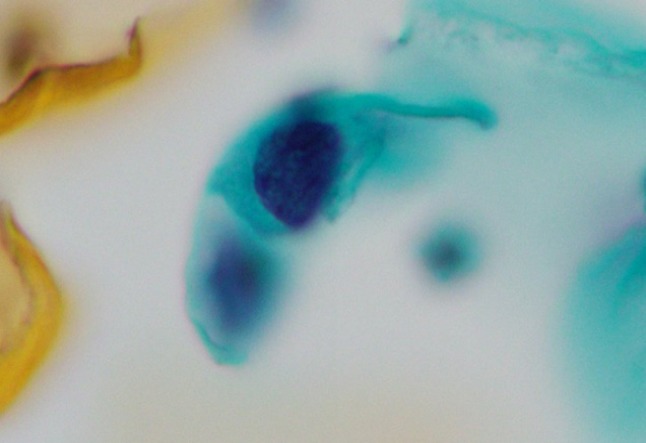
Smear Showing ASC-H with Marked Hyperchromatic Cells with High Nuclear/Cytoplasmic Ratio (Pap Staining, ×40)

**Figure 2:**
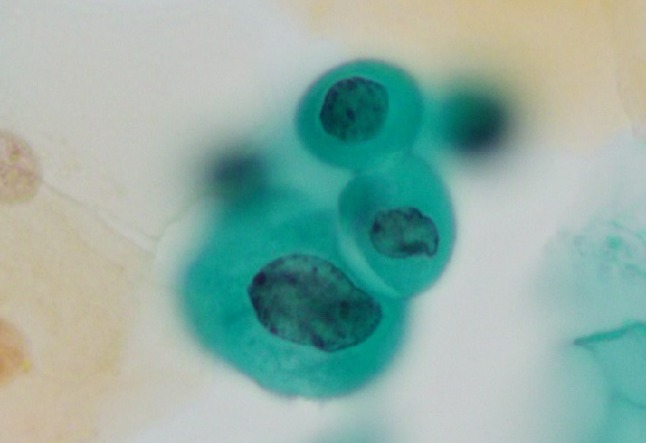
Smear Showing ASC-H with Thickening of Nuclear Contour with High Nuclear/Cytoplasmic Ratio and Irregular Nuclear Memblane (Pap Staining ×40)

**Figure 3:**
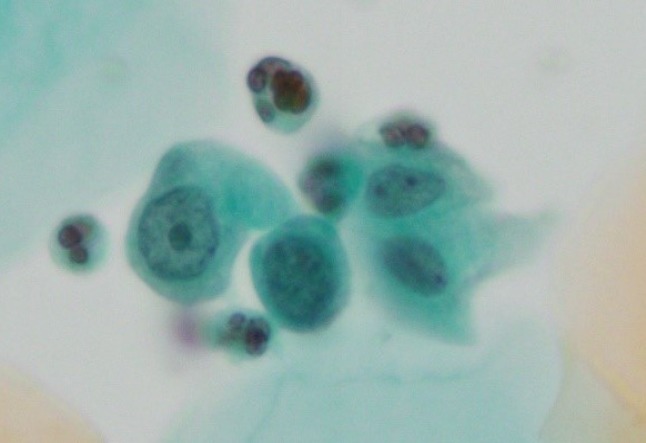
Smear Showing ASC-H with Prominent Nucleoli with High Nuclear/Cytoplasmic Ratio (Pap Saining, ×40)

Mean age of cases (n = 913) was 37.0 years (range 20–56 years). Of the 913 cases, 25 exhibited ASC-H on Pap smear, which was reviewed and confirmed by a pathologist. Of the 25 ASC-H cases, 22 (88%) were cervical intraepithelial neoplasia-positive (CIN+). The biopsies performed for these 25 cases showed the following results: benign lesions, 3 cases (1 atypical metaplasia and 2 chronic cervicitis); CIN grade 1 (CIN1+) lesions, 3 cases; CIN grade 2 (CIN2+) lesions, 9 cases; and CIN grade 3 (CIN3+) lesions, 10 cases (Table 1). HPV was detected in 66.7% (2/3) of benign cases and in 100% (22/22) of CIN1+ cases. The total number of ASC-H in this study was 247 in Table 1. The correlation between cytological findings of ASC-H and histopathological diagnosis is shown in Table 2. ASC-H liquid-based Pap smears with biopsy-proven CIN1+ showed dense cytoplasmic differentiation in 155 (71.4%) cells, marked hyperchromasia in 129 (59.4%) cells (Figure 1), thickening of nuclear contour in 169 (77.9%) cells (Figure 2), and one or more prominent nucleoli in 73 (33.6%) cells (Figure 3).

**Table 1:** Histological Diagnosis, HPV Genotypes, and the Number of ASC-H in the Pap Smear and Concurrent Punch Biopsy

Case No.	Histological diagnosis/Grade	HPV genotypes	The number of ASC-H cells per preparation
1	Benign	-	4
2	Benign	56	12
3	Benign	16, 52, 53	14
4	CIN1	16, 33	7
5	CIN1	16	1
6	CIN1	52, 68	6
7	CIN2	16	3
8	CIN2	52	7
9	CIN2	16	7
10	CIN2	51	3
11	CIN2	51, 52, 84	14
12	CIN2	52	19
13	CIN2	31	13
14	CIN2	33, 52, 68, 30	5
15	CIN2	18	8
16	CIN3	58	15
17	CIN3	67	17
18	CIN3	16, 52, 55, 71	7
19	CIN3	16, 69	13
20	CIN3	16	3
21	CIN3	16	16
22	CIN3	16	26
23	CIN3	52	15
24	CIN3	31	2
25	CIN3	58	10

CIN, cervical intraepithelial neoplasia; ASC-H, atypical squamous cells; cannot exclude high-grade squamous intraepithelial lesion.

**Table 2:** Correlation between Cytological findings of ASC-H Cells and the Concurrent Histological Diagnosis

	Cytoplasmic differentiation		Chromatin				Nuclear/Cytoplasmic ratio		Nuclear shape irregularity	Thickening of nuclear contour	Prominent nucleoli
	Dense	Pale	Hyper chromasia not marked	Marked hyperchromasia	Fine	Coarse	Low	High			
Benign 30 cells	90.0% (27)	10.0% (3)	70.0% (21)	30.0% (9)	96.7% (29)	3.3% (1)	43.3% (13)	56.7% (17)	3.3% (1)	43.3% (13)	6.7% (2)
CIN1+ 217 cells	71.4% (155)	28.6% (62)	40.6% (88)	59.4% (129)	94.5% (205)	5.5% (12)	43.8% (95)	56.2% (122)	1.8% (4)	77.9% (169)	33.6% (73)

CIN, cervical intraepithelial neoplasia

In contrast, benign cases showed dense cytoplasmic differentiation in 27 (90.0%) cells, marked hyperchromasia in 9 (30.0%) cells, thickening of nuclear contour in 13 (43.3%) cells, and prominent nucleoli in 2 (6.7%) cells. Marked hyperchromasia (p < 0.01), thickening of nuclear contour (p < 0.01), and prominent nucleoli (p < 0.01) were significantly different between benign and CIN1+ lesions. 

## Discussion

Morphological analysis of concurrently obtained cytology and histology specimens demonstrated a diverse pattern in the cytological criteria for ASC-H diagnosis based on the Bethesda system. Significant differences were observed in chromatin pattern, thickening of nuclear contour, and nucleoli between benign and CIN1+ lesions. Reportedly, the diversity of the cells is one of the reasons for difficulty in judging ASC-H (Louro et al., 2003), but this diversity maybe the key criteria for the existence of CIN. In the follow-up studies on the cytomorphological features of ASC-H, compared with benign cases, the cells in CIN cases have been found to exhibit a higher N/C ratio, hyperchromasia, coarse chromatin, and irregular nuclear margins (Chivukula and Shidham, 2006; Alli and Ali, 2008). However, these cytomorphological features are also the criteria for the diagnosis of HSIL, as described in the Bethesda system, and they only highlight the cytological features of ASC-H, which by nature is “not to exclude HSIL.” Furthermore, the reliability on individual (cytotechnologist and pathologist) judgment for defining a “higher N/C ratio” comparing ASC-H to benign cells causes difficulty in defining ASC-H cytomorphological features.

It is widely known that thickening of nuclear contour is observed in cells with herpes simplex virus (HSV) infection. A study has shown that HSV particles cause partial thickening of the nuclear margin (Wada and Kimura, 1985). Another study has reported the presence of HPV particles in the nuclear membrane in the vicinity of irregularly condensed chromatin in condyloma patients. (Casas-Cordero et al., 1981; Kera et al., 1987). Although HSV was not detected in this study, high-risk HPV was detected in all CIN1+ cases. Therefore, it is possible that HPV also causes nuclear marginal thickening of squamous epithelial cells through the same mechanism as HSV.

We further ruled out the presence of glandular cells, which share the findings of a prominent nucleoli and thickening of nuclear contour. Therefore, when prominent nucleoli and thickening of nuclear contour are seen on Pap smear, caution must be exercised before interpreting the findings as reactive glandular derived cells, as the presence of a prominent nucleolus has been reported to be associated with benign cases (Chivukula and Shidham, 2006; Alli and Ali, 2008; Mokhtar et al., 2008). In this study, CIN+ ASC-H exhibited prominent nucleoli and showed statistical significance in an analysis of all small atypical cells of each sample. We believe that this statistical significance was found because we evaluated every single atypical cell, instead of randomly selecting traditionally representative cells of ASC-H.

The fact that only a few benign cases were analyzed in this study limits the generalizability of the results, and therefore, larger sample sizes are necessary to further elucidate the findings of this study. 

In conclusion, the presence of small dysplastic cells with cytomorphological features such as marked hyperchromasia, thickening of nuclear contour, and prominent nucleoli on Pap smear of ASC-H cases may strongly suggest the presence of CIN.
